# MYC-family protein overexpression and prominent nucleolar formation represent prognostic indicators and potential therapeutic targets for aggressive high-MKI neuroblastomas: a report from the children’s oncology group

**DOI:** 10.18632/oncotarget.23740

**Published:** 2017-12-15

**Authors:** Risa Niemas-Teshiba, Ryosuke Matsuno, Larry L. Wang, Xao X. Tang, Bill Chiu, Jasmine Zeki, Jeannine Coburn, Kimberly Ornell, Arlene Naranjo, Collin Van Ryn, Wendy B. London, Michael D. Hogarty, Julie M. Gastier-Foster, A. Thomas Look, Julie R. Park, John M. Maris, Susan L. Cohn, Robert C. Seeger, Shahab Asgharzadeh, Naohiko Ikegaki, Hiroyuki Shimada

**Affiliations:** ^1^ Department of Pathology and Laboratory Medicine, Children’s Hospital Los Angeles, University of Southern California Keck School of Medicine, Los Angeles, CA 90027, USA; ^2^ Department of Anatomy and Cell Biology, College of Medicine, University of Illinois at Chicago, Chicago, IL 60612, USA; ^3^ Department of Surgery, College of Medicine, University of Illinois at Chicago, Chicago, IL 60612, USA; ^4^ Department of Biomedical Engineering, Worcester Polytechnic Institute, Worcester, MA, 01609, USA; ^5^ Department of Biostatistics, Children’s Oncology Group Statistics and Data Center, University of Florida, Gainesville, FL 32607, USA; ^6^ Division of Hematology/Oncology, Boston Children’s Hospital and Dana-Farber Cancer Institute, Harvard Medical School, Boston, MA 02215, USA; ^7^ Division of Oncology and Department of Pediatrics, Children’s Hospital of Philadelphia, University of Pennsylvania School of Medicine, Philadelphia, PA 19104, USA; ^8^ Institute of Genomic Medicine, Nationwide Children’s Hospital and Departments of Pathology and Pediatrics, Ohio State University College of Medicine, Columbus, OH 43210, USA; ^9^ Department of Pediatric Oncology, Dana-Farber Cancer Institute, Harvard Medical School, Boston, MA 02215, USA; ^10^ Department of Pediatrics, Seattle Children’s Hospital, University of Washington School of Medicine and Fred Hutchinson Cancer Research Center, Seattle, WA 98105, USA; ^11^ Department of Pediatrics, Division of Hematology/Oncology, University of Chicago, Chicago, IL 60637, USA; ^12^ Division of Hematology/Oncology, Children’s Hospital Los Angeles, University of Southern California Keck School of Medicine, Los Angeles, CA 90027, USA; ^13^ Department of Pediatrics, Showa University Fujigaoka Hospital, Yokohama 1–30, Japan; ^14^ Department of Biology, Loyola University Chicago, Chicago, IL 60660, USA

**Keywords:** nucleolar hypertrophy, protein translation, aminoacyl-tRNA synthetase, halofuginone, CX-5461

## Abstract

Neuroblastomas with a high mitosis-karyorrhexis index (High-MKI) are often associated with *MYCN* amplification, MYCN protein overexpression and adverse clinical outcome. However, the prognostic effect of MYC-family protein expression on these neuroblastomas is less understood, especially when *MYCN* is not amplified. To address this, MYCN and MYC protein expression in High-MKI cases (120 *MYCN* amplified and 121 non-*MYCN* amplified) was examined by immunohistochemistry. The majority (101) of *MYCN*-amplified High-MKI tumors were MYCN(+), leaving one MYC(+), 2 both(+), and 16 both(−)/(+/−), whereas non-*MYCN*-amplified cases appeared heterogeneous, including 7 MYCN(+), 36 MYC(+), 3 both(+), and 75 both(−)/(+/−) tumors. These MYC-family proteins(+), or MYC-family driven tumors, were most likely to have prominent nucleolar (PN) formation (indicative of augmented rRNA synthesis). High-MKI neuroblastoma patients showed a poor survival irrespective of *MYCN* amplification. However, patients with MYC-family driven High-MKI neuroblastomas had significantly lower survival than those with non-MYC-family driven tumors. MYCN(+), MYC-family protein(+), PN(+), and clinical stage independently predicted poor survival. Specific inhibition of hyperactive rRNA synthesis and protein translation was shown to be an effective way to suppress MYC/MYCN protein expression and neuroblastoma growth. Together, MYC-family protein overexpression and PN formation should be included in new neuroblastoma risk stratification and considered for potential therapeutic targets.

## INTRODUCTION

Peripheral neuroblastic tumors (pNTs including neuroblastoma, ganglioneuroblastoma, and ganglioneuroma) represent one of the most common extra-cranial neoplasms in children [1]. They are composed of biologically distinct groups of tumors whose genomic/molecular characteristics can directly be associated with a wide range of clinical behaviors in individual patients from a benign or non-aggressive course largely due to spontaneous regression or tumor maturation to an extremely aggressive course often resulting in fatal outcome. International efforts have focused on development of the most appropriate protocols for managing pNT patients by stratifying them according to the defined risk grouping systems based on the combination of various prognostic factors [2]. For example, the Children’s Oncology Group (COG) Neuroblastoma Classification Biology Study, ANBL00B1, has been utilizing Age at Diagnosis [3], Clinical Staging (International Neuroblastoma Staging System: INSS) [4], Histopathology (International Neuroblastoma Pathology Classification: INPC) [5–8], *MYCN* oncogene amplification status [9, 10], DNA index [11], and Chromosomal aberrations [12] for assigning Low, Intermediate, or High Risk protocol to pNT patients from North America, Australia, and New Zealand [13].

The INPC, one of the prognostic factors critically contributing to the COG risk grouping, was established in 1999 [5, 6] and modified in 2003 [8]. It includes four morphologic indicators: Schwannian stromal development (distinguishing Neuroblastoma category, Ganglioneuroblastoma, Intermixed category, and Ganglioneuroma category), Grade of neuroblastic differentiation [distinguishing Undifferentiated (NB-U), Poorly differentiated (NB-PD), and Differentiating (NB-D) subtypes in Neuroblastoma category], Mitosis-Karyorrhexis index [MKI: distinguishing Low (< 100/5,000 cells), Intermediate (100–200/5,000 cells) and High (> 200/5,000 cells) class in Neuroblastoma category], and grossly visible neuroblastomatous nodule formation co-existing with Ganglioneuroblastoma, Intermixed or Ganglioneuroma (identifying Ganglioneuroblastoma, Nodular category). Among these morphologic indicators, there is a reproducible relationship between *MYCN* amplification and increased MKI classes [14, 15]: Our recent study confirmed that 68% of High-MKI, 15% of Intermediate-MKI, and only 3% of Low-MKI neuroblastomas had *MYCN* oncogene amplification [16].

*MYCN* oncogene amplification has been recognized as the strongest indicator of aggressive tumor behavior for pNT patients. Recently we reported that MYCN protein overexpression after DNA amplification could be the critical step in bringing the aggressive phenotype to pNTs [17]. Our latest study further showed that MYCN protein overexpression was detected in ∼20% of patients with neuroblastoma of undifferentiated (NB-U) and poorly differentiated (NB-PD) subtypes [18]. We also discovered that MYC (aka C-myc) protein overexpression was observed in another ∼10% of patients in the same cohort. Because these two subsets of neuroblastomas showed almost identical survival curves, we have combined them together and initially termed them MYC-driven neuroblastoma [18]. However, to avoid the confusion among physicians and researchers in the field, we have renamed this highly aggressive subset of neuroblastomas as “MYC family protein-driven neuroblastomas” (or MYC family-driven neuroblastomas in short). While investigating these MYC family-driven neuroblastomas, we recognized that their tumor cells were often associated with the presence of prominent nucleoli.

*MYC* family genes are among the most frequently activated genes in human cancer [19] and their activation leads to the elevated expression of their protein products. MYC family proteins include MYC, MYCN and MYCL, and they are known to play pivotal roles in development, differentiation, cell growth, cell death, stem cell self-renewal and probably more. MYC family proteins are basic-helix-loop-helix-leucine zipper transcription factors that activate the expression of MYC responsive genes. To activate their target genes, MYC family proteins first bind to their obligatory partner protein called MAX and form heterodimers. These active forms of MYC family proteins then bind to the specific DNA sequence, called E-Boxes. Notably, there are numerous canonical and non-canonical E-box sequences with varying affinities in the genome, and thus cells with high levels of MYC family protein expression tend to occupy more E-boxes than those with low MYC family protein expression. Upon the specific DNA binding, MYC family proteins recruit the scaffold protein, TRRAP, to the sites [19]. This molecular complex further recruits histone acetyl transferases such as GCN5. Therefore, high MYC family protein expression subsequently results in a genome-wide increase in histone acetylation and ultimately high contents of euchromatin [20].

MYC family proteins are known to activate many genes involved in rRNA synthesis, protein translation and mitochondriogenesis, glucose and glutamine metabolism, and lipid synthesis [21]. Therefore cells with high levels of MYC family protein expression tend to show hypertorophy of the cell body [22, 23], nuclei [20] and nucleoli [24]. These cellular changes brought by the augmented MYC family protein expression ultimately contribute to malignant transformation of the cells.

In this study, we focused on a subset of unfavorable neuroblastomas with a High-MKI and analyzed the pattern of MYC and MYCN protein expression compared to the distribution of conventional prognostic factors based on their *MYCN* oncogene status (amplified vs. non-amplified). We further examined a possible relationship between prominent nucleolar formation and augmented expression of MYC family proteins. Then we performed survival analyses to test prognostic significance of MYC family protein expression, prominent nucleolar formation, and other conventional prognostic factors. We also analyzed their prognostic relationships in this cohort of High-MKI neuroblastoma patients. Lastly, we tested the feasibility of small molecule inhibitors that specifically target hyperactive rRNA synthesis and augmented protein translation suppressing growth and elevated expression of MYC family proteins in unfavorable neuroblastoma cells.

## RESULTS

**Figure 1:**
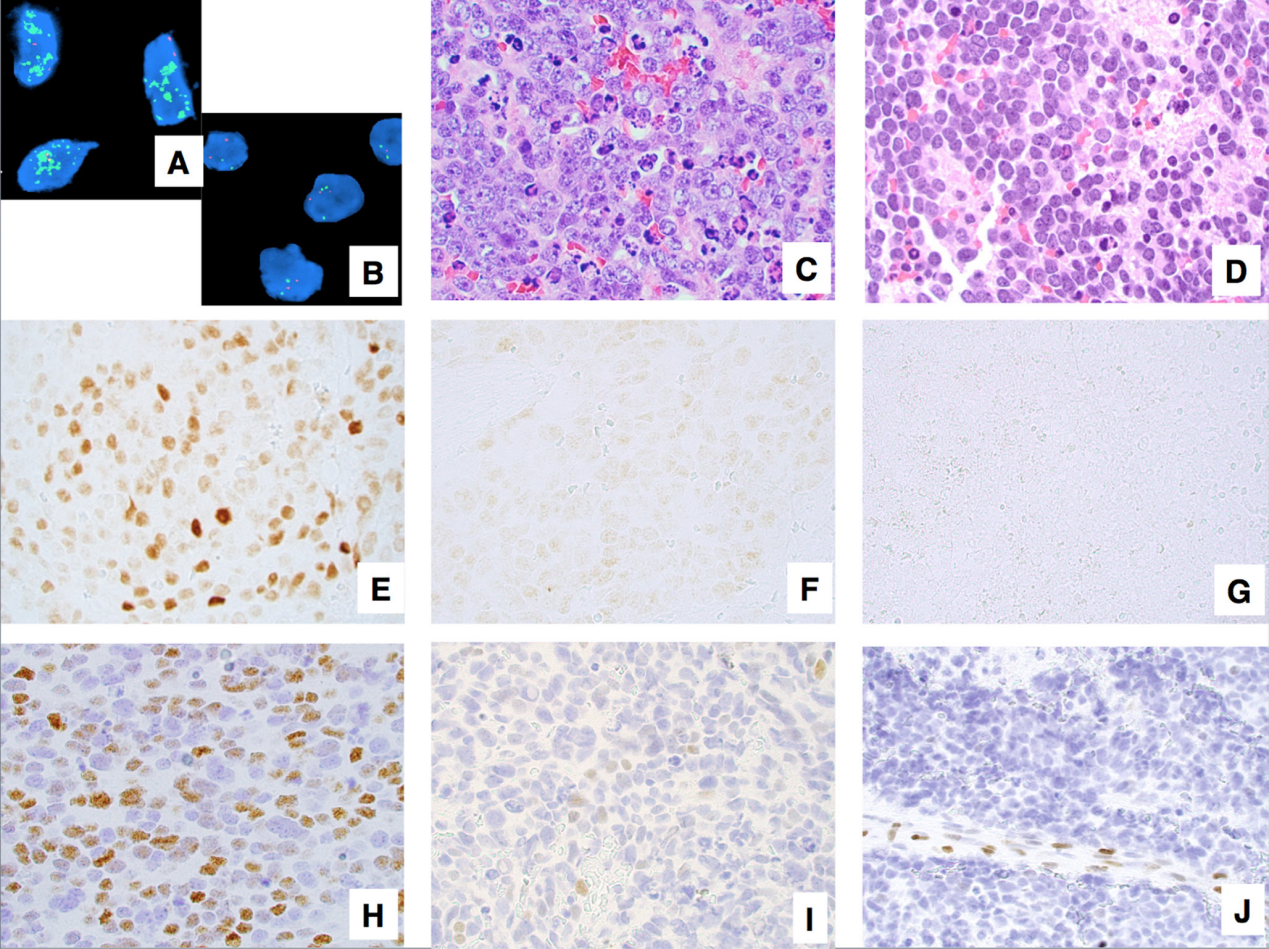
Examples of neuroblastoma, undifferentiated and poorly differentiated subtype with a High Mitosis-Karyorrhexis Index (**A)** Tumor in the *MYCN* amplified group (FISH, Original × 1,000) Green = 2p23-24 *MYCN* region probe; Red = *CEP 2* region probe. (**B**) Tumor in the *MYCN* non-*MYCN* amplified group (FISH, Original × 1,000) Green = 2p23-24 *MYCN* region probe; Red = *CEP 2* region probe. (**C**) Tumor with prominent nucleolar formation (H&E, Original x600). (**D**) Tumor without prominent nucleolar formation (H&E, Original x600). (**E**) Tumor expressing MYCN protein (+) (Immunostaining without counterstaining, Original × 600). (**F**) Tumor with MYCN protein (+/−) (Immunostaining without counterstaining, Original × 600). (**G**) Tumor with MYCN protein (−) (Immunostaining without counterstaining, Original × 600). (**H**) Tumor expressing MYC protein (+) (Immunostaining with Hematoxylin counterstaining, Original × 600). (**I**) Tumor with MYC protein (+/−) (Immunostaining with Hematoxylin counterstaining, Original x600). (**J**) Tumor with MYC protein (−) Note activated endothelial cells positive for MYC protein (Immunostaining with Hematoxylin counterstaining, Original × 600).

In this study, we analyzed a total of 241 High-MKI cases including 120 *MYCN* amplified (NB-U 28, NB-PD 92) and 121 non-*MYCN* amplified (NB-U 4, NB-PD 117) tumors (Figure 1A and 1B), all of which had available H&E slides and unstained slides in the file of COG Neuroblastoma Pathology Reference Laboratory (see details in Materials and Methods).

### Pathology review

For those 241 cases, H&E slides were re-reviewed by RN-T, RM, LW, and HS, according to the International Neuroblastoma Pathology Classification (INPC). The median number of slides reviewed was 3.80 (range 1 to 26) per case. Diagnostic criteria of INPC were previously described [5, 6]. High-MKI tumors were defined when the number of mitotic and karyorrhectic cells per 5,000 denominator cells was more than 200 [5]. Definition of prominent nucleolar formation is described in our previous reports [17, 25]. In the prominent nucleoli (+) tumors, 10% or more of neuroblastic cells had one or a few large prominent nucleoli (Figure 1C). In the prominent nucleoli (−) tumors, a majority of neuroblastic cells had conventional salt-and-pepper pattern of nuclei (none or less than 10% of neuroblastic cells with prominent nucleoli, Figure 1D).

The results of immunostaining were classified as follows (see Figure 1): negative (−); focal or sporadic, and weak nuclear staining (+/−); and diffuse and strong nuclear staining with typically heterogeneous intensity (+). However, in our previous study, the survival effect for negative (−) and focal or sporadic, and weak (±) staining were similar, therefore we combined both groups together for further analyses. Accordingly each tumor was determined to have either MYCN protein overexpression [MYCN protein (+)] or MYCN protein negative/weak expression [MYCN protein (−)/(+/−)] (Figures 1E, 1F, and 1G) and either MYC protein overexpression [MYC protein (+)] or MYC protein negative/weak expression [MYC protein (−)/(+/−)] (Figures 1H, 1I, and 1J). Appropriate positive and negative controls were stained along with those tumors.

### Clinicopathological characteristics of High MKI Neuroblastomas based on MYCN oncogene status

**Figure 2:**
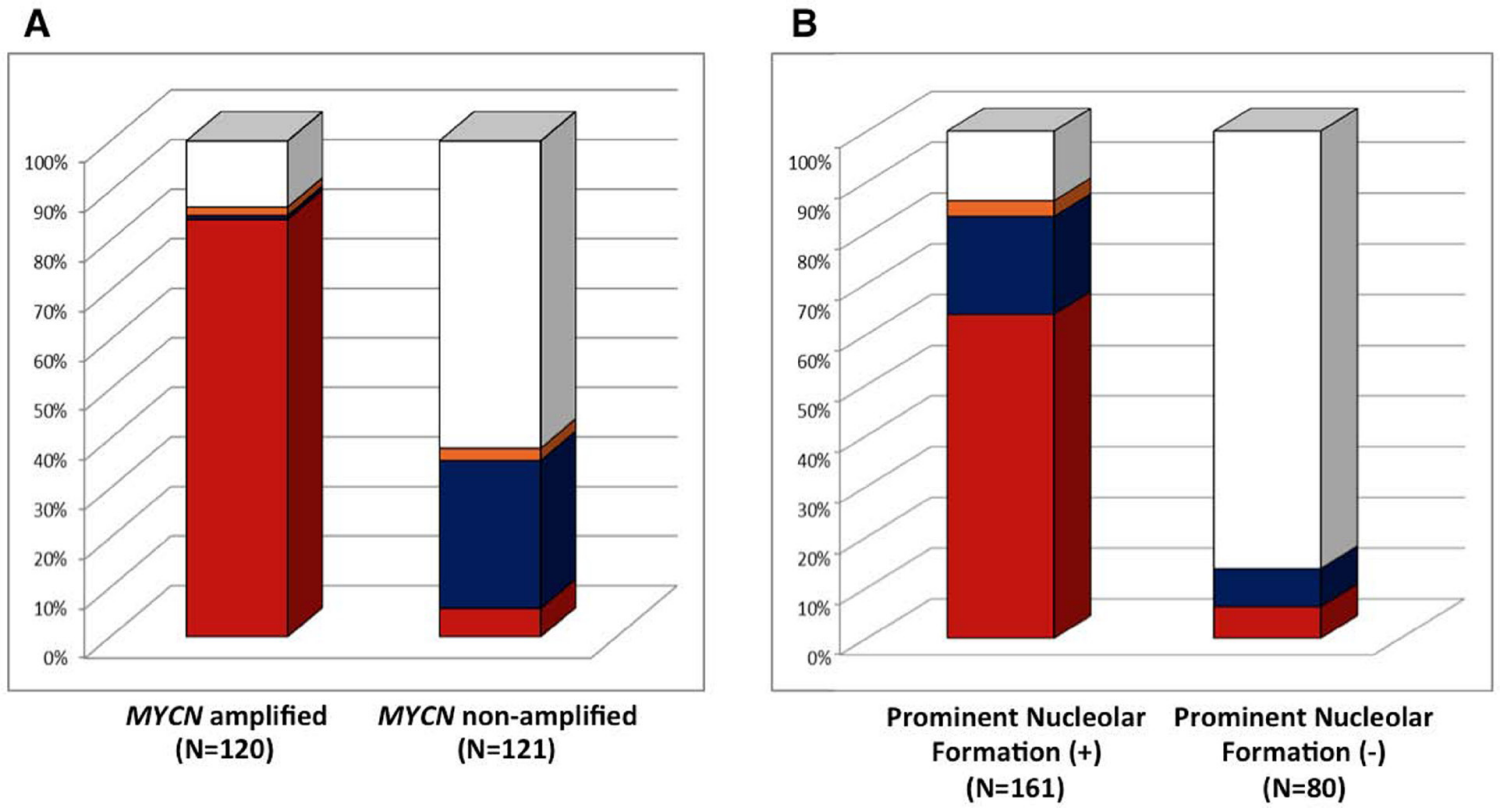
Neuroblastoma, undifferentiated and poorly differentiated subtype with a High Mitosis-Karyorrhexis Index **(A**) Protein Expression by *MYCN* status: Among *MYCN* amplified group, 101 (84.2% red) expressed MYCN protein (+) only, one (0.8% blue) expressed MYC protein (+) only, 2 (1.7% orange) expressed both proteins (+), and 16 (13.3% white) were both proteins (−)/(+/−). Among non-*MYCN* amplified group, 7 (5.8% red) expressed MYCN protein (+) only, 36 (29.8% blue) expressed MYC protein (+) only, 3 (2.5% orange) expressed both proteins (+), and 75 (62.0% white) were both proteins (−)/(+/−). (**B**) Protein Expression and Prominent Nucleolar Formation: Among Prominent Nucleolar Formation (+) group, 103 (64.0% red) expressed MYCN protein (+) only, 31 (19.3% blue) expressed MYC protein (+) only, 5 (3.1% orange) expressed both proteins (+), and 22 (13.7% white) were both proteins (−)/(+/−). Among Prominent Nucleolar Formation (−) group, 5 (6.3% red) expressed MYCN protein (+) only, 6 (7.5% blue) expressed MYC protein (+) only, and 69 (86.3% white) were both proteins (−)/(+/−).

Among *MYCN* amplified High-MKI cases (*n* = 120), 101 (84.2%) tumors were MYCN protein (+), one (0.8%) tumor was MYC protein (+), 2 tumors (1.7%) were both proteins (+), and 16 tumors (13.3%) were both proteins (−)/(+/−). In contrast, non-*MYCN* amplified High-MKI cases (*n* = 121) included 7 (5.8%) MYCN protein (+) tumors, 36 (29.8%) MYC protein (+) tumors, 3 (2.5%) both proteins (+) tumors, and 75 (62.0%) both proteins (−)/(+/−) tumors (see Figure 2A).

Controlling for *MYCN* amplification (see Table 1A, 1B), there were no significant differences in the distributions of conventional prognostic factors (Age at diagnosis, DNA Ploidy pattern, INSS clinical stage, and Grade of neuroblastic differentiation) between MYCN protein (+) vs. both proteins (−)/(+/−), MYC protein (+) vs. both proteins (−)/(+/−), and either protein (+) vs. both proteins (−)/(+/−).

**Table 1A:** *MYCN* amplified group: Prominent nucleolar formation and prognostic factors by MYCN protein and MYC protein expression

MYCN-P	MYC-P	PN		Age		Ploidy		INSS Stage		Grade	
		(−)	(+)	< 18 months	≥ 18 months	Hyper-diploid	Diploid	Non-stage 4	Stage 4	U	PD
(+)	any	3	98	42	59	33	60	23	78	25	76
(−)/(+/−)	(−)/(+/−)	8	8	7	9	8	6	3	13	3	13
*p*-value		**< 0.0001**		0.8704		0.1202		1.0000		0.7586	
any	(+)	0	3	0	3	1	2	0	3	0	3
(−)/(+/−)	(−)/(+/−)	8	8	7	9	8	6	3	13	3	13
*p*-value		0.2281		0.2632		0.5765		1.0000		1.0000	
Either (+)		3	101	42	62	34	62	23	81	25	79
(−)/(+/−)	(−)/(+/−)	8	8	7	9	8	6	3	13	3	13
*p*-value		**< 0.0001**		0.7988		0.1180		1.0000		0.7607	

**Table 1B:** Non *MYCN*-amplified group: Prominent nucleolar formation and prognostic factors by MYCN protein and MYC protein expression

MYCN-P	MYC-P	PN		Age		Ploidy		INSS Stage		Grade	
		(−)	(+)	< 18 months	≥18 months	Hyper-diploid	Diploid	Non-stage 4	Stage 4	U	PD
(+)	any	2	5	1	6	3	4	1	6	0	7
(−)/(+/−)	(−)/(+/−)	61	14	25	50	46	26	24	51	2	73
*p*-value		**0.0064**		0.4223		0.4171		0.4306		1.0000	
any	(+)	6	33	12	27	23	13	13	26	2	37
(−)/(+/−)	(−)/(+/−)	61	14	25	50	46	26	24	51	2	73
*p*-value		**< 0.0001**		0.7815		1.0000		0.8853		0.6053	
Either (+)		8	38	13	33	26	17	14	32	2	44
(−)/(+/−)	(−)/(+/−)	61	14	25	50	46	26	24	51	2	73
*p*-value		**< 0.0001**		0.5595		0.7135		0.8571		0.6344	

MYCN-P = MYCN protein; MYC-P = MYC protein; PN = Prominent nucleolar formation; Age = Age at diagnosis; Ploidy = DNA ploidy pattern; INSS = International Neuroblastoma Staging System; Grade = Grade of neuroblastic differentiation; U = Undifferentiated; PD = Poorly differentiated.

There were, however, significant differences in the distribution of prominent nucleolar formations between all the three comparison groups within the non-*MYCN* amplified tumors. In each case, tumors with both proteins (−)/(+/−) were less likely to have prominent nucleolar formations. Within the *MYCN* amplified tumors, there were significant differences in distribution of prominent nucleolar formations between MYCN protein (+) vs. both (−)/(+/−), and either protein (+) vs. both (−)/(+/−), but not between MYC protein (+) vs. both (−)/(+/−) [Note: there were only 3 tumors with *MYCN* amplification and MYC protein (+)]. In both cases, MYC family-driven neuroblastomas with MYCN protein (+) or either protein (+) were more likely to have prominent nucleoli.

When the *MYCN* amplified and non-*MYCN* amplified High-MKI cases were combined (*n* = 241), 161 tumors were prominent nucleolar formation (+) and 139 (86.3%) of them were MYC family-driven neuroblastomas with MYCN protein (+), MYC protein (+), or both (+), as shown in Figure 2B. Eighty tumors were prominent nucleolar formation (−) and the majority of them [69 (86.2%)] did not have MYCN protein (+) and MYC protein (+). In summary, there was a significant relationship between MYCN/MYC protein overexpression and prominent nucleolar formation in this group of tumors (*p* < 0.0001).

### Survival analysis

**Figure 3:**
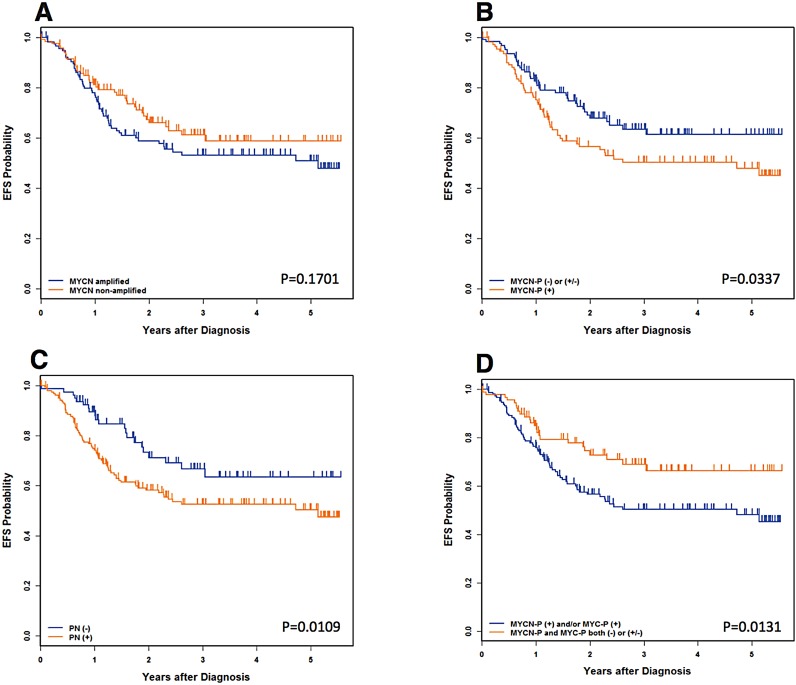
Neuroblastoma, undifferentiated and poorly differentiated subtype with a High Mitosis-Karyorrhexis Index (**A**) Event-Free Survival by *MYCN* oncogene status. (**B**) Event-Free Survival by MYCN Protein Expression: MYCN-P = MYCN protein. (**C**) Event-Free Survival by Prominent nucleolar formation: PN = prominent nucleoli. (**D**) Event-Free Survival by MYCN/MYC Protein Expression: MYCN-P = MYCN protein, MYC-P = MYCN protein. Please see Table 2A and 2B for the data including the number of the patients, 4-year Event-Free survival, and 4-year Overall survival.

The median follow-up time for patients without event was 2.7 years. The median follow-up time for patients who did not die was 2.8 years. The 4-year event-free survival (EFS) and overall survival (OS) estimates for all 241 patients were 56.2+/−5.5% and 64.5+/−5.4%, respectively. As shown in Table 2A (all patients) and Figure 3, univariate analysis showed significant differences in survival between MYCN protein (+) and MYCN protein (−)/(+/−) patients, with more favorable outcomes for MYCN protein (−)/(+/−) patients. Significant differences in survival were also detected between prominent nuclear formation (+) and (−), with more favorable outcomes for prominent nucleolar formation (−) patients.

**Table 2A:** EFS and OS for all patients by *MYCN* oncogene status, MYCN-P status, and MYC-P status

Cohort	Number	4-year EFS ± SE (%)	EFS *p*-value	4-year OS ± SE (%)	OS *p*-value
Overall	241	56.2 ± 5.5	N/A	64.5 ± 5.4	N/A
*MYCN* Non-amplified Amplified	121 (50%) 120 (50%)	58.8 ± 10.9 53.2 ± 6.3	0.1701	70.7 ± 9.9 59.3 ± 6.3	0.0913
MYCN-P (−)/(+/−) (+)	128 (53%) 113 (47%)	61.4 ± 9.9 50.4 ± 6.5	**0.0337**	73.8 ± 8.9 55.9 ± 6.5	**0.0096**
MYCN-P by *MYCN* Non-amplified, (−)/(+/−) Non-amplified, (+) Amplified, (−)/(+/−) Amplified, (+)	111 (46%) 10 (4%) 17 (7%) 103 (43%)	59.1 ± 11.4 52.5 ± 36.2 76.9 ± 18.5 50.0 ± 6.6	0.1601	72.4 ± 10.2 39.4 ± 30.7 84.6 ± 16.6 56.3 ± 6.6	0.0656
MYC-P (−)/(+/−) (+)	199 (83%) 42 (17%)	57.0 ± 5.8 51.0 ± 17.8	0.9244	65.0 ± 5.7 58.0 ± 16.8	0.7212
MYC-P by *MYCN* Non-amplified, (−)/(+/−) Non-amplified, (+) Amplified, (−)/(+/−) Amplified, (+)	82 (34%) 39 (16%) 117 (49%) 3 (1%)	62.5 ± 12.8 50.2 ± 20.5 53.2 ± 6.4 50.0 ± 35.4	0.4793	76.2 ± 11.2 55.5 ± 18.5 58.8 ± 6.4 100.0 ± 0.0	0.2312
MYCN-P, MYC-P Both (−)/(+/−) Either or Both (+)	91 (38%) 150 (62%)	66.3 ± 11.1 50.2 ± 6.2	**0.0131**	80.1 ± 9.5 56.1 ± 6.1	**0.0064**

**Table 2B:** EFS and OS by MYCN-P and MYC-P status, controlling for *MYCN* amplification

Cohort		Number	4-year EFS ± SE (%)	EFS *p*-value	4-year OS ± SE (%)	OS *p*-value
*MYCN* amplified						
MYCN-P	MYC-P					
(+) (−)/(+/−)	(−)/(+/−) (−)/(+/−)	101 (86%) 16 (14%)	50.0 ± 6.7 76.9 ± 18.5	0.1111	55.6 ± 6.7 84.6 ± 16.6	0.0898
*MYCN* amplified						
MYCN−P (+) and/or MYC−P (+) Both proteins (−)/(+/−)		104 (87%) 16 (13%)	50.0 ± 6.6 76.9 ± 18.5	0.1139	56.3 ± 6.6 84.6 ± 16.6	0.0965
Non-*MYCN* amplified						
MYCN-P	MYC-P					
(+) (−)/(+/−) (−)/(+/−)	(−)/(+/−) (+) (−)/(+/−)	7 (6%) 36 (31%) 75 (64%)	34.3 ± 27.8 48.3 ± 20.1 64.1 ± 13.6	0.1176	34.3 ± 27.8 54.4 ± 18.4 79.4 ± 11.4	0.0750
Non-*MYCN* amplified						
MYCN−P (+) and/or MYC−P (+) Both proteins (−)/(+/−)		46 (38%) 75 (62%)	49.1 ± 17.5 64.1 ± 13.6	0.1192	51.2 ± 16.0 79.4 ± 11.4	0.1095

EFS = Event-free survival; OS = Overall survival; SE = standard error; MYCN-P = MYCN protein; MYC-P = MYC protein

Survival of patients with MYC family-driven neuroblastomas where the tumors had MYCN protein (+) and/or MYC protein (+) was significantly worse than that of patients with non-MYC family-driven neuroblastomas where both proteins were (−)/(+/−). Four-year EFS for MYC family-driven High-MKI neuroblastomas was 50.2+/−6.2% and that for non-MYC family-driven High-MKI neuroblastomas was 66.3+/−11.1% (*p* = 0.0131; Table 2A). Four-year OS for MYC-family High-MKI neuroblastomas was 56.1+/−6.1% and that for non-MYC family-driven protein driven High-MKI neuroblastomas was 80.1+/−9.5% (*p* = 0.0064).

No other survival comparisons yielded significant results, including *MYCN* amplified vs. non-*MYCN* amplified patients and controlling for *MYCN* status (Table 2B). Some statistical tests were hampered by small sample sizes or, for the outcome comparisons, a lack of events. Such groups include: non-*MYCN* amplified and MYCN protein (+), *MYCN* amplified and MYCN protein (−)/ (+/−), and *MYCN* amplified and MYC protein (+).

### Prognostic relationship among prominent nucleolar formation, MYC family protein expression and other conventional prognostic indicators

A full backward-selected Cox model in terms of EFS and OS including prominent nucleolar formation was fit, allowing any covariate to drop out of the model. The final Cox model indicated that prominent nucleolar formation [(+) vs. (−)] and INSS stage (stage 4 vs. non-stage 4) were both predictive of EFS and OS (Table 3A). A full backward-selected Cox model in terms of EFS and OS including MYCN protein was fit, allowing any covariate to drop out of the model. The final Cox model indicated that MYCN protein [(+) vs. (−)/(+/−)] and INSS stage (stage 4 vs. non-stage 4) were predictive of both EFS and OS (Table 3B). A full backward-selected Cox model in terms of EFS and OS, including MYC protein, was fit allowing any covariate to drop out of the model. The final Cox model indicated that only INSS stage (stage 4 vs. non-stage 4) was predictive of survivals for EFS and OS (Table 3C).

Finally, a full backward-selected Cox model for EFS and OS including MYC family protein expression, i.e. both proteins (−)/(+/−) vs. either protein (+), was fit, allowing any covariate to drop out of the model. The final Cox model indicated that INSS stage and MYC family protein expression were both predictive of survival for EFS and OS (Table 3D). The order of removal was *MYCN* amplification, Age at diagnosis, DNA ploidy, and Grade of neuroblastic differentiation for EFS; Age at diagnosis, *MYCN* amplification, Grade of neuroblastic differentiation, and DNA ploidy for OS, with the least statistically significant term dropping out at each step. While controlling for INSS stage, the model predicted that patients who expressed MYC family proteins were at 1.870 times greater risk of an event than patients who expressed neither protein (*p* = 0.0087) and at 2.245 times greater risk of death than patients who expressed neither protein (*p* = 0.0045). In essence, MYC family protein expression retained its prognostic significance in the presence of standard neuroblastoma risk factors in the Cox models for both EFS and OS in High MKI neuroblastomas, and it had a better predictability of adverse outcome than MYCN protein expression alone.

**Table 3A:** Final backward-selected Cox model testing PN and prognostic factors

		< Event-free survival >				< Overall survival >		
Variable (*N* = 241)	DF	*p*-value	Hazard	95% CI on	DF	*p*-value	Hazard	95% CI on
			Ratio	Hazard Ratio			Ratio	Hazard Ratio
INSS Stage (non-stage 4^*^ vs. stage 4)	1	0.0002	3.377	(1.794, 6.355)	1	0.0003	4.208	(1.942, 9.206)
PN [(−)^*^ vs. (+)]	1	0.0113	1.881	(1.154, 3.065)	1	0.0083	2.201	(1.226, 4.952)

**Table 3B:** Final backward-selected Cox model testing MYCN-P and prognostic factors

		< Event-free survival >				< Overall survival >		
Variable (*N* = 241)	DF	*p*-value	Hazard	95% CI on	DF	*p*-value	Hazard	95% CI on
			Ratio	Hazard Ratio			Ratio	Hazard Ratio
MYCN-P [(−)/(+/−)^*^ vs. (+)]	1	0.0441	1.533	(1.011, 2.325)	1	0.0145	1.822	(1.127, 2.948)
INSS Stage (non-stage 4^*^ vs. stage 4)	1	0.0003	3.214	(1.709, 6.044)	1	0.0005	3.994	(1.826, 8.734)

**Table 3C:** Final backward-selected Cox model testing MYC-P and prognostic factors

		< Event-free survival >				< Overall survival >		
Variable (*N* = 241)	DF	*p*-value	Hazard	95% CI on	DF	*p*-value	Hazard	95% CI on
			Ratio	Hazard Ratio			Ratio	Hazard Ratio
INSS Stage (non-stage 4^*^ vs. stage 4)	1	0.0003	3.231	(1.718, 6.074)	1	0.0005	4.019	(1.839, 8.784)

**Table 3D:** Final backward-selected Cox model testing MYCN-P/MYC-P and prognostic factors

		< Event-free survival >				< Overall survival >		
Variable (*N* = 241)	DF	*p*-value	Hazard	95% CI on	DF	*p*-value	Hazard	95% CI on
			Ratio	Hazard Ratio			Ratio	Hazard Ratio
MYCN-P/MYC-P both (−)/(+/−)^*^ vs. either (+)	1	0.0087	1.87	(1.172, 2.983)	1	0.0045	2.245	(1.285, 3.920)
INSS Stage (non-stage 4^*^ vs. stage 4)	1	0.0002	3.33	(1.769, 6.266)	1	0.0004	4.16	(1.901, 9.102)

EFS = Event-free survival; OS = Overall survival; PN = Prominent nucleolar formation; INSS = International Neuroblastoma Staging System; DF = Degree of freedom; MYCN-P = MYCN protein; MYC-P = MYC protein

^*^Indicates the reference level for each variable. The hazard ratio is the increased risk of an event in comparison to this reference level.

### Inhibition of overdriven protein synthesis by inhibitors of RNA Pol I and an aminoacyl-tRNA synthetase results in MYC/MYCN down regulation in MYC family-driven neuroblastoma cells

Our results show that prominent nucleolar formation (nucleolar hypertrophy), indicative of overdriven rRNA gene transcription as well as protein translation, could provide critical prognostic and risk-stratification information for neuroblastoma. At the same time, this observation could open up an opportunity for innovative therapy for aggressive neuroblastomas. To assess this possibility, we examined the effect of two potent and specific small molecule inhibitors of RNA Pol I activity and an aminoacyl tRNA synthetase (CX-5461 [26] and Halofuginone [27], respectively) on growth and MYC family protein expression in neuroblastoma cells.

**Figure 4:**
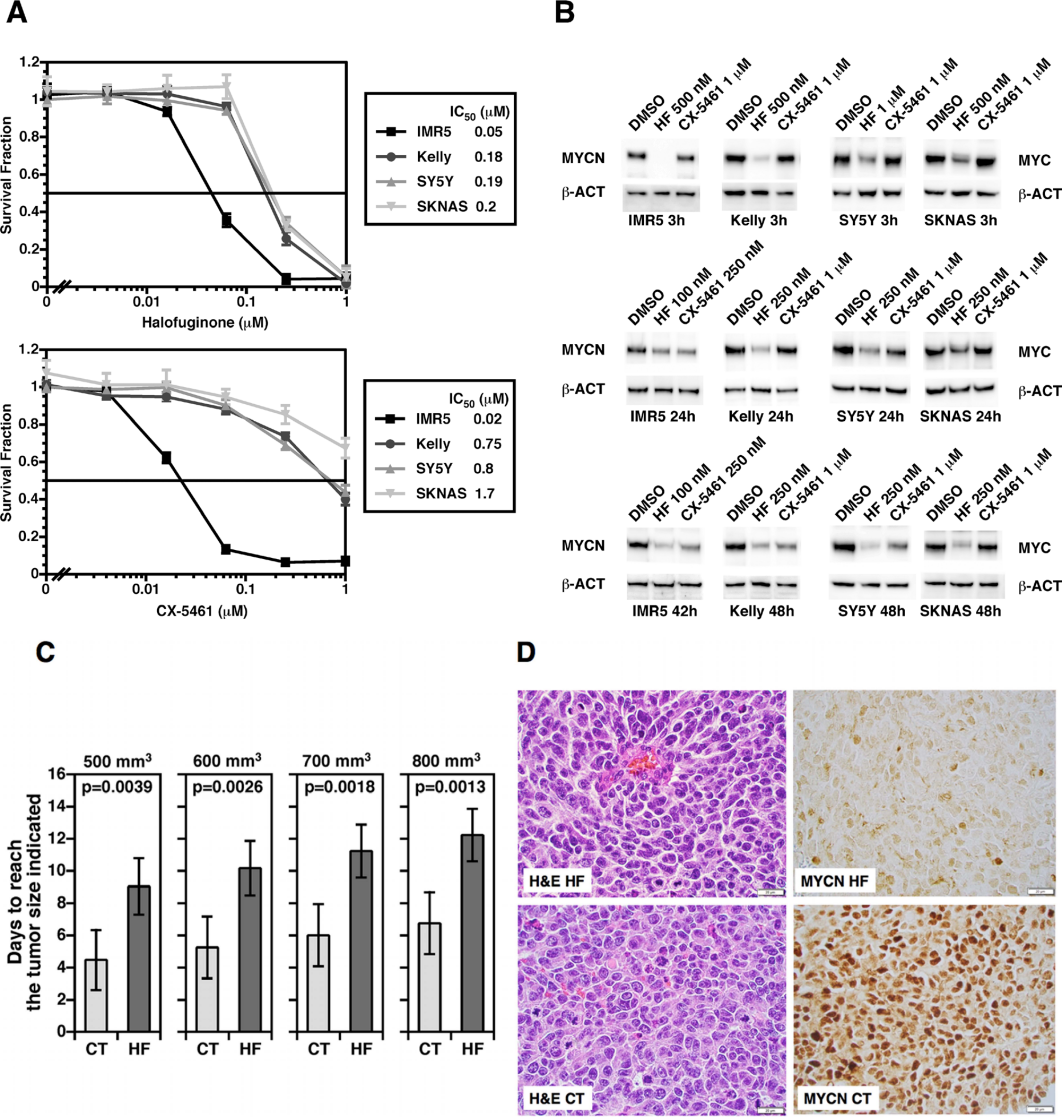
(**A**) Halofuginone (an inhibitor of glutamyl-prolyl-tRNA synthetase) and CX-5461 (an inhibitor of rRNA synthesis) significantly suppress growth of neuroblastoma cells *in vitro*. Four neuroblastoma cell lines with various genetic profiles were treated with these small molecule inhibitors at the concentrations indicted for 48 hours. IMR5 is *MYCN* amplified and *TP53* normal; Kelly is *MYCN* amplified and *TP53* mutated; SY5Y is non-*MYCN* amplified and *TP53* normal; SKNAS is non-*MYCN* amplified and *TP53* mutated. At the end of the 48 hour-incubation, cell were subjected the MTS assay to determine growth effect of the compounds on the neuroblastoma cells. (**B**) Halofuginone and CX-5461 down-regulate MYC and MYCN expression in neuroblastoma cells at low to submicromolar concentrations. Western blot assay employing anti-pan MYC monoclonal antibody (NCM II 143) was used to determine the effect of the compounds on MYC and MYCN protein expression in neuroblastoma cells. Beta-actin (β-ACT) was used as a protein loading control. (**C**) The preclinical efficacy of Halofuginone on growth of Kelly orthotopic xenografts. One million Kelly cells per mouse were implanted into the left adrenal grand of nude mice. When the tumors reached > 100 mm3, silk films loaded with 20 μg Halofuginone (HF; *n* = 5) or PBS/DMSO (CT; *n* = 5) were surgically inserted into the tumors. Growth of tumors was monitored by high frequency ultrasound using a VisualSonics Vevo 2100 sonographic probe (Toronto, Ontario, Canada), and the tumor volume was measured using the 3-D reconstruction tool (Vevo Software v1.6.0, Toronto, Ontario, Canada). Statistical analysis was done using a Student *t*-test. (**D**) The effect of Halofuginone on MYCN expression in Kelly xenografts. Kelly xenografts treated with the HF silk films (HF) or control silk films (CT) were stained with H&E (H&E HF vs. H&E CT) or processed for MYCN immunohistochemistry MYCN HF vs. MYCN CT). Xenografts were resected at 17 days after the initiation of HF treatment (20 μg).

As shown in Figure 4A, these small molecule inhibitors in fact inhibited growth of neuroblastoma cell lines with various genetic profiles at low to submicromolar concentrations *in vitro* in forty-eight hours. Moreover, the inhibitors destabilized MYC and MYCN proteins in neuroblastoma cells (Figure 4B). Interestingly, Halofuginone showed a rapid effect on the stability of MYC family proteins at 500 nM-1 µM (< 3 hours). In contrast, CX-5461 at 1 mM was less effective at the 3-hour time. At the concentration of 100 nM-250 nM for Halofuginone and 1 µM for CX-5461 for up to forty-eight hours of the drug treatments, MYC and MYCN expression were both down-regulated, though SKNAS was less responsive to CX-5461 than other cell lines. It should be mentioned that both compounds are among the most effective small molecules that can suppress the expression of MYC family proteins. There appeared to be no apparent correlation between the drug response and the genetic profiles of cell lines used in the experiment.

### Inhibition of glutamyl-prolyl-tRNA synthetase by Halofuginone suppresses growth of orthotopic Kelly neuroblastoma xenografts in mice and MYCN expression

**Figure 5:**
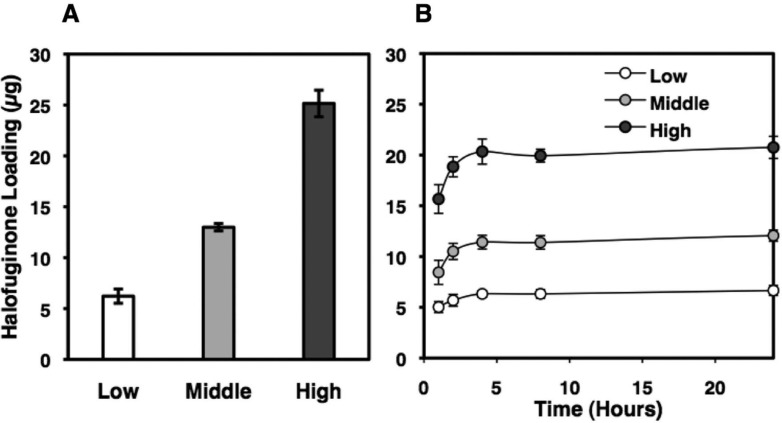
Halofuginone-loading in and release from silk films Silk films were loaded with Halofuginone via adsorption from solution method as described in the methods section. (**A**) The resulting Halofuginone loading masses were 6 ± 0.7 μg (low), 13 ± 0.4 μg (middle), 25 ± 1.3 μg (high). (**B**) Halofuginone release in PBS was monitored via UV spectroscopy (314 nm). All of the Halofuginone release from the low and middle loading groups, and 86% of the Halofuginone release from the high group.

Next, we examined the preclinical efficacy of Halofuginone in orthotopic Kelly human neuroblastoma xenografts in mice using a silk film as the drug delivery device. Since Halofuginone has a better theoretical drug retention and release profile from the silk film than CX-5461 (see below), we tested Halofuginone as a single drug in this drug delivery model in this study. As shown in Figure 4C, Halofuginone showed a significant growth suppressive effect on the xenografts, as indicated by the time periods to reach certain tumor sizes being significantly longer in the Halofuginone treated group than those in the control group. Moreover, histological examination of the xenografts showed a marked suppression of MYCN protein expression in the Halofuginone treated xenograft at day 17 of the drug treatment. Since Halofuginone (20 µg) was released from the silk film over the initial 24-hour period (Figure 5), the down regulation of MYCN in response to Halofuginone appeared to be sustained for a long time period.

## DISCUSSION

This report examined MYCN and MYC protein expression and prominent nucleolar formation in High-MKI neuroblastomas and assessed the contribution of these factors to survival of this unfavorable cohort of patients in relation to the conventional prognostic factors, including *MYCN* amplification. As expected, the vast majority (103/120, 85.8%) of *MYCN* amplified High-MKI neuroblastomas overly expressed MYCN protein, including 2 tumors also having simultaneous MYCN and MYC protein expression. In contrast, more than 60% of non-*MYCN* amplified High-MKI neuroblastomas were MYCN protein (−)/(+/−) and MYC protein (−)/(+/−). It is interesting to note that MYC protein overexpression was associated with around 30% of High MKI neuroblastomas without *MYCN* amplification. In our previous study, MYC protein overexpression was shown to be associated more with rather Low- or Intermediate-MKI than High-MKI in neuroblastomas [18]. However, this study demonstrated that MYC protein overexpression could also represent a considerable proportion of non-*MYCN* amplified-High-MKI tumors. It is also noted that there were some non-*MYCN* amplified-High-MKI tumors (*N* = 10, 8.3%) that overly expressed MYCN protein with or without simultaneous MYC protein expression. This observation suggests that certain mechanisms exist to allow overexpression or stabilization of MYCN protein without gene amplification, such as ones described in other reports [28, 29].

Tests comparing the distributions of conventional prognostic factors, such as age at diagnosis, DNA ploidy, INSS clinical stage, and grade of neuroblastic differentiation, showed no significant difference between *MYCN* amplified and non-*MYCN* amplified groups of High-MKI neuroblastomas. However, there were significant differences in the presence of prominent nucleolar formation in both *MYCN* amplified and non-amplified groups, and prominent nucleolar formation was more likely to be present in MYC family-driven neuroblastomas overly expressing MYCN and/or MYC protein. These results confirmed our previous findings using a different cohort of patients [18, 30]. The observation is also consistent with the facts that ribosomal RNA gene repeats organize into nucleoli [31] and that their transcription is regulated directly and indirectly by MYC family proteins [24, 32]. It is of interest to note that in this series of High-MKI neuroblastomas, there were a total of 22 tumors with prominent nucleolar formation which did not express excess amount of MYCN or MYC protein. Further investigations will be needed to assess the involvement of other proteins, such as MYCL [33–35], in association with prominent nucleolar formation.

According to the INPC, High MKI is a sign of poor prognosis regardless of age at diagnosis for neuroblastoma [5, 6, 16]. Notably however, *MYCN* amplification status was not predictive of survival in this cohort. The factors shown to be predictive of outcome in this cohort of High-MKI neuroblastomas were whether or not tumors expressed augmented levels of MYC family proteins or if they exhibited prominent nucleolar formation. It is also noted that patients with MYC family-driven High-MKI neuroblastomas had a significantly lower EFS and OS than patients with non-MYC family-driven High-MKI neuroblastomas. When we analyzed prognostic relationship among the conventional prognostic factors, MYC family protein expression and prominent nucleolar formation, INSS stage, MYCN protein expression, the combined MYC family protein expression and prominent nucleolar formation were the only factors that independently predicted clinical outcome of High-MKI neuroblastoma patients.

Nuclear morphology of neuroblastic cells is often described as a “salt-and-pepper” appearance. However, as shown in this study as well as in our previous studies, there are clinically aggressive MYC family-driven neuroblastomas whose tumor cells are often characterized by the presence of one or a few prominent nucleoli as a sign of nucleolar hypertrophy. Morphological changes of the nucleus and its substructures associated with tumor aggressiveness often reflect genetic, epigenetic and biological changes. In order to exhibit nucleolar hypertrophy, indicative of hyper-activated ribosomal DNA transcription, cells need to open chromatin at ribosomal RNA gene repeats. The ribosomal RNA gene repeats then have to be transcriptionally activated by the complex of necessary transcription factors and RNA Polymerase I (Pol I). MYC-family proteins, after forming the heterodimer with the partner MAX protein, play pivotal roles in the process of ribosomal DNA transcription: the MYC/MAX complex binds E-boxes near ribosomal RNA genes and recruits histone acetyl transferases, allowing open chromatin configuration [36]. The MYC/MAX complex can also up-regulate the expression of upstream binding transcription factor (UBTF) and Pol I-specific transcription initiation factor RRN3, which are obligatory transcription factors in ribosomal RNA gene expression [37]. Therefore, the emergence of prominent nucleoli in tumor cells most likely suggests elevated expression of MYC family proteins or their functional equivalents.

It has been suggested that selective inhibition of rRNA gene transcription in the nucleolus by small molecule inhibitors could be an effective therapeutic strategy for malignant tumors [37, 38]. On the other hand, augmented protein translation, which would link to hyperactive rRNA synthesis, could be suppressed by specific inhibition of various steps involved in translation. In this study, we examined the feasibility of such strategies in neuroblastoma. CX-5461 is a small molecule that disrupts the binding of the SL1 transcription factor to the rDNA promoter and prevents the initiation of rRNA synthesis by RNA Pol I [26]. Consequently, ribosomal assembly will be halted, leading to the accumulation of unassembled ribosomal proteins [37, 38]. Free ribosomal proteins will then promote cancer-specific activation of p53 [37, 38]. Interestingly, a similar set of free ribosomal proteins could also down-regulate MYC protein by distinct mechanisms [39, 40]. Accordingly, our study showed that CX-5461 down-regulated MYC and MYCN proteins and suppressed growth of neuroblastoma cells.

Considering the inhibitory activity on RNA Pol I transcription by CX-5461, one may be concerned with severe side effects of the drug. However, the preclinical evaluation of CX- 5461 by Drygin et al. showed that CX-5461 was well-tolerated at all tested schedules as judged by the absence of significant changes in animal body weights [26]. Thus, it is expected that side effects of CX-5461 would be low, if any. In addition, it has been reported that in a Phase I study (ACTRN12613001061729), CX-5461 is well tolerated with low-grade manageable adverse events to date. Inhibition of Pol I transcription in peripheral blood mononuclear cells indicated on-target drug activity in the initial dose cohorts. This trial is continuing in dose escalation [41]. Another Phase I/II Study of CX-5461 is currently on going (https://clinicaltrials.gov/ct2/show/NCT02719977?term=CX-5461&rank=1) and these trials will answer this question in the near future.

Halofuginone specifically inhibits glutamyl-prolyl tRNA synthetase (EPRS). This results in the accumulation of uncharged prolyl tRNAs, which then leads to suppression of protein translation via the induction of the amino acid starvation response [42]. In addition, inhibition of EPRS may also liberate one of the cofactors from the human multi-tRNA-synthetase complex (i.e., AIMP2). Freed AIMP2 could then migrate into the nucleus and inhibit FBP (FUBP1), which otherwise functions as the transcriptional activator of *MYC*. Consequently, Halofuginone could induce down regulation of MYC family proteins through both global translational suppression and transcriptional inhibition. Our study, for the first time, showed that Halofuginone effectively down-regulated MYC and MYCN proteins in MYC family driven neuroblastoma cells.

As about half of “prominent nucleolar formation (+)” or “MYC family protein-driven High-MKI” tumors fail the current super intensive therapy, inhibition of ribosomal RNA gene transcription and aminoacyl-tRNA synthetases by small molecule inhibitors [26, 27] could be a potential therapeutic approach for this subset of unfavorable neuroblastomas as our results suggest (Figure 4). To this end, both CX-5461 and Halofuginone are currently examined for their efficacy on various human diseases in clinical trials (ACTRN12613001061729; https://clinicaltrials.gov/ct2/show/NCT02719977; https://clinicaltrials.gov/ct2/show/NCT02525302?term=halofuginone&rank=3).

Finally, results of this study along with recently accumulated data clearly prompt us to develop a new scheme of pathology classification as well as risk stratification for patients with neuroblastoma by incorporating the information on the expression of MYC family proteins in conjunction with nuclear morphology of the tumor cells. Moreover, MYC-family protein overexpression and prominent nucleolar formation should be considered for potential therapeutic targets of aggressive unfavorable neuroblastomas.

## MATERIALS AND METHODS

### Patient cohort

Between January 1, 2009 and June 18, 2014 a total of 3,280 pNTs were reviewed at the COG Neuroblastoma Pathology Reference Laboratory, Department of Pathology and Laboratory Medicine, Children’s Hospital Los Angeles, Los Angeles, California. Of these, 2,605 tumors were in the Neuroblastoma (Schwannian stroma-poor) category and included the undifferentiated subtype (NB-U, 85 cases), the poorly differentiated subtype (NB-PD, 2,321 cases), and the differentiating subtype (NB-D, 199 cases). In order to avoid potential confusion in identifying/detecting unique prominent nucleoli, those NB-D cases were excluded from the study, as, by definition, differentiating neuroblasts in this subtype had one prominent nucleolus as a sign of nuclear maturation. As for the NB-U and NB-PD cases, MKI classes were determined in 2,366 tumors: 1,234 were Low MKI (NB-U 5, NB-PD 1045), 660 were Intermediate MKI (NB-U 19, NB-PB 628), and 472 were High MKI (NB-U 56, NB-PD 415). Among the High-MKI cases, *MYCN* status was determined by FISH test in 448 tumors, and results were reported as “Amplified (309 tumors; NB-U 51, NB-PD 258)” and “Non-amplified (139 tumors; NB-U 4, NB-PD 135)”. Informed consent approved by the institutional review board was obtained for all patients at the time of enrollment in the COG biology or therapeutic study.

### Immunohistochemistry

Unstained sections from the study cases were heated for 30 minutes in Bond™ Epitope Retrieval Solution 2 (No. AR9640; Leica Biosystems Newcastle Ltd, Benton Ln, Newcastle Upon Tyne, UK) using Leica BOND-MAX™ (Leica Microsystems Inc, Bannockburn, IL, USA), and incubated with either anti-MYCN mouse monoclonal antibody (NCM II 100 [43] at a dilution of 1:200) or anti-human MYC rabbit monoclonal antibody (clone Y69; No. #1472–1; Epitomics, Cambridge, MA, USA at a dilution of 1:200) in Bond™ Primary Antibody Diluent (No. AR9352; Vision BioSystems Inc., Norwell, MA, USA). The counter staining with hematoxylin was performed for MYC protein staining slides, but no counter staining was performed for MYCN protein staining slides.

### Statistical analysis

First, we compared *MYCN* amplified and non*-MYCN* amplified High-MKI tumors based on their protein expression pattern. Then, controlling for *MYCN* amplification, the distribution of prognostic factors, such as presence of prominent nucleolar formation [PN (+) vs. PN (−)], Age at diagnosis (< 18 months vs. ≥ 18 months), INSS stage (non-stage 4 vs. stage 4), DNA ploidy pattern (hyperdiploid vs. diploid), and Grade of neuroblastic differentiation (NB-U vs NB-PD), were compared with chi-squared, or if small samples, Fisher’s exact tests, between the following groups: MYCN protein (+) vs. both proteins (−)/(+/−), MYC protein (+) vs. both proteins (−)/(+/−), and either protein (+) vs. both proteins (−)/(+/−).

Event-free survival (EFS) was calculated as the number of days from diagnosis to the occurrence of an event or, if no event, the date of last follow-up. An event is defined as death, disease relapse or progression, or secondary malignancy. Overall survival (OS) was calculated as the number of days from diagnosis to death or, if the patient did not die, the date of last follow-up. Kaplan-Meier EFS and OS estimates with standard errors per the methods of Peto et al. [44, 45] were computed and log-rank tests were performed to compare EFS and OS between the groups by *MYCN* oncogene status, prominent nucleolar formation, and MYCN/MYC protein expression status. Also analyzed were EFS and OS by MYCN/MYC protein expression status controlling for *MYCN* oncogene amplification status. Survival plots and life tables were produced. Survival probabilities are presented as 4-year estimates ± standard error, written as percentages. *P*-values less than 0.05 were considered statistically significant.

To determine the independent prognostic strength for survival of the protein expression factors in the presence of the prognostic factors; age at diagnosis, INSS stage, *MYCN* amplification status, DNA ploidy pattern, grade of neuroblastic differentiation, and prominent nucleolar formation, Cox proportional hazards (PH) models with the Efron method of handling tied event times were fit. Backward selection was used to determine the most parsimonious model.

### Neuroblastoma cell growth assay and Western blot assay *in vitro*

*MYCN* amplified cell lines, IMR5 and Kelly, and non-*MYCN* amplified cell lines, SY5Y and SKNAS, were used in this study. IMR5 and SY5Y have normal *TP53* gene, whereas Kelly and SKNAS cells harbor mutations in *TP53* gene. IMR5 and Kelly cells express high levels of MYCN protein, whereas SY5Y and SKNAS cells express high levels of MYC protein. IMR5, a clone of IMR32, was a gift of Roger H. Kennett (University of Pennsylvania, PA. USA) who derived this clone. Kelly and SY5Y cell lines were obtained from Sigma (St. Louis, MO, USA). SKNAS cell line was a gift of Susan L. Cohn (University of Chicago, IL, USA). Growth of these neuroblastoma cell lines in response to CX-5461 and Halofuginone was assessed by MTS assay as described in our previous study [46]. Neuroblastoma cells were treated with the small molecule inhibitors at the concentrations and durations indicated in Figure 4. Cells were then subjected to Western blot assay using anti-pan-MYC antibody, NCM II 143 [43] and anti-β-Actin antibody (Santa Cruz) as described in our previous studies [47, 48].

### Mouse orthotopic neuroblastoma model

All mouse procedures were performed in accordance with University of Illinois’ recommendations for the care and use of animals and were maintained and handled under protocols approved by the Institutional Animal Care and Use Committee. All procedures were performed with female NCr nude mice (Taconic, Hudson, NY, USA) at 7 weeks of age. Procedures and ultrasound measurements (see below) were performed under general anesthesia using isoflurane inhalation. Orthotopic tumors within the adrenal gland were created as described in our previous study [49]. Briefly, a transverse incision was made on the left flank to locate the left adrenal gland, and 2 mL of phosphate buffered saline (PBS) containing 10^6^ Kelly cells were injected into the adrenal gland using a 30G needle. The fascia and skin were closed in separate layers. Tumor formation was monitored by non-invasive ultrasound measurements, and the animals euthanized when the tumor volume exceeded 1,000 mm^3^.

### Monitoring tumor growth by high frequency ultrasound

After securing the mouse in a prone position, a VisualSonics Vevo 2100 sonographic probe (Toronto, Ontario, Canada) was applied to the left flank to locate the left adrenal gland and the tumor. Serial cross-sectional images (0.076 mm between images) were taken. The tumor volume was measured using the 3-D reconstruction tool (Vevo Software v1.6.0, Toronto, Ontario, Canada).

### Silk fibroin extraction

Silk fibroin from *Bomby Mori* silkworm cocoons from Tajima Shoji Co (Yokohama, Japan) was extracted as previously described [50]. Briefly, 5 g of cocoons were cut into approximately 1 cm × 1 cm pieces and boiled in 0.02 M Na_2_CO_3_ for 30 minutes to extract the silk fibroin. After drying overnight, the extracted silk fibroin was dissolved in 9.3 M LiBr at 60°C for 3 hrs. The dissolved silk fibroin was dialyzed against deionized water for 2 days. The aqueous silk fibroin solutions was stored at 4°C for future use.

### Silk film fabrication

Silk films were fabricated as previously described [49, 51, 52]. Briefly, 192 μL of 4% (w/v) silk fibroin was pipetted onto 11 cm × 17 cm polydimethylsiloxane (Sylgard^®^184, Dow Corning, Auburn, MI) and allowed to dry overnight. Seven millimeter diameter disks were cut from the dried silk films and autoclaved to induce the β-sheet transition and render the materials insoluble.

### Halofuginone loading and drug release studies

Halofuginone-loaded silk films were formulated via an adsorption from solution method [51, 52]. Silk films (1.2 ± 0.04 mg) were loaded with Halofuginone via adsorption presumably through electrostatic interaction between the positively charge Halofuginone (basic pKa = 9.28; ChemAxon, Cambridge, MA) and negatively charge silk fibroin (isoelectric points ranging from 4.0 – 5.2 [53]). Halofuginone was dissolved in dimethyl sulfoxide at 50 mg/mL. Films were submerged in 1 mL of 31 µg/mL, 16 µg/mL and 8.5 µg/mL Halofuginone solution (diluted in sterile deionozed water) for 2 days. Halofuginone concentration was determined via UV/visible spectroscopy at 314 nm using a standard curve method (ranging from 1.6 µg/mL to 200 µg/mL). The Halofuginone loading was calculated as follows:

$$\left(\mathrm{preadsorption}\;\mathrm{concentration}\left(\frac{\mu g}{\mathrm{ml}}\right)-\mathrm{postadsorption}\;\mathrm{concentration}\left(\frac{\mu g}{\mathrm{ml}}\right)*1\mathrm{mL}\right)=\mathrm{Halofuginone}\;\mathrm{Loading}\left(\mu g\right)$$

For drug release studies, Halofuginone-loaded silk films were placed into 0.5 mL phosphate buffered saline (pH7.4) at 37°C in a 1.5 mL Eppendorf tube. The absorbance of the PBS solution was read at each time point and solution replaced into the Eppendorf tube for study continuation. The PBS was not changed during the experimental time course to maintain the concentration above the minimal detectable concentration (∼ 2 µg/mL). For drug release studies, silk films were loaded with 25 ± 1.3 µg, 13 ± 0.4 µg and 6 ± 0.7 µg Halofuginone (Figure 5A). Halofuginone exhibited sustained release over the first 24 hours (Figure 5B).

## Abbreviations

PN: prominent nucleolar or prominent nucleolus
rRAN: ribosomal RNA
